# Identification of diagnostic genes for both Alzheimer’s disease and Metabolic syndrome by the machine learning algorithm

**DOI:** 10.3389/fimmu.2022.1037318

**Published:** 2022-11-02

**Authors:** Jinwei Li, Yang Zhang, Tanli Lu, Rui Liang, Zhikang Wu, Meimei Liu, Linyao Qin, Hongmou Chen, Xianlei Yan, Shan Deng, Jiemin Zheng, Quan Liu

**Affiliations:** ^1^ Department of Neurosurgery, The Fourth Affiliated Hospital of Guangxi Medical University, Liuzhou, China; ^2^ General Surgery, The First Affiliated Hospital of Dali University, Dali, China; ^3^ Department of Neurology, The Tenth Affiliated Hospital of Guangxi Medical University, Qinzhou, China; ^4^ College of Bioengineering, Chongqing University, Chongqing, China; ^5^ Department of Neurology, The Fourth Affliated Hospital of Guangxi Medical University, Liuzhou, Guangxi, China

**Keywords:** Alzheimer’s disease, metabolic syndrome, XGBost, machine learning algorithm, immune infiltration, single cell sequencing

## Abstract

**Background:**

Alzheimer’s disease is the most common neurodegenerative disease worldwide. Metabolic syndrome is the most common metabolic and endocrine disease in the elderly. Some studies have suggested a possible association between MetS and AD, but few studied genes that have a co-diagnostic role in both diseases.

**Methods:**

The microarray data of AD (GSE63060 and GSE63061 were merged after the batch effect was removed) and MetS (GSE98895) in the GEO database were downloaded. The WGCNA was used to identify the co-expression modules related to AD and MetS. RF and LASSO were used to identify the candidate genes. Machine learning XGBoost improves the diagnostic effect of hub gene in AD and MetS. The CIBERSORT algorithm was performed to assess immune cell infiltration MetS and AD samples and to investigate the relationship between biomarkers and infiltrating immune cells. The peripheral blood mononuclear cells (PBMCs) single-cell RNA (scRNA) sequencing data from patients with AD and normal individuals were visualized with the Seurat standard flow dimension reduction clustering the metabolic pathway activity changes each cell with ssGSEA.

**Results:**

The brown module was identified as the significant module with AD and MetS. GO analysis of shared genes showed that intracellular transport and establishment of localization in cell and organelle organization were enriched in the pathophysiology of AD and MetS. By using RF and Lasso learning methods, we finally obtained eight diagnostic genes, namely *ARHGAP4*, *SNRPG*, *UQCRB*, *PSMA3*, *DPM1*, *MED6*, *RPL36AL* and *RPS27A*. Their AUC were all greater than 0.7. Higher immune cell infiltrations expressions were found in the two diseases and were positively linked to the characteristic genes. The scRNA-seq datasets finally obtained seven cell clusters. Seven major cell types including CD8 T cell, monocytes, T cells, NK cell, B cells, dendritic cells and macrophages were clustered according to immune cell markers. The ssGSEA revealed that immune-related gene (*SNRPG*) was significantly regulated in the glycolysis-metabolic pathway.

**Conclusion:**

We identified genes with common diagnostic effects on both MetS and AD, and found genes involved in multiple metabolic pathways associated with various immune cells.

## Introduction

Alzheimer’s disease (AD) is the most common age-related neurodegenerative disease. Its process is slow, chronic and fatal, which challenges the world’s medical care. The slowdown in the progression of AD may be the greatest unmet medical need of our time (1). Similar to Parkinson’s disease, clinical symptoms of AD appear, its pathophysiological changes have been existing and developing for decades. Therefore, a better understanding of the mechanisms behind AD to identify new biomarkers for early diagnosis, treatment and prognosis is urgently needed.

Metabolic syndrome (MetS) is a general term for risk factors of cardiovascular and cerebrovascular diseases, diabetes, obesity and hypertension (2), including insulin resistance, low level good cholesterol (HDL), abdominal obesity, hypertension and hypertriglyceridemia, which affects approximately 35% of adults (3). MetS is not only a health problem, but also an economic burden. Clinical and epidemiological evidence indicates that MetS clusters such as obesity, hypertension, dyslipidemia and Type 2 diabetes promote the development of mild cognitive impairment (MCI), dementia and AD in several ways (4–8). A meta-analysis of 9,788,021 patients with an average follow-up of 4.5 years showed a significant association between MetS and AD (9). MetS can activate microglia through the interface of the blood-brain barrier (2). A growing body of epidemiological evidence has allowed the development of a pathophysiological model called “metabolic-cognitive syndrome”, which aims to understand the complex relationship between metabolic disorders and cognitive impairment, thereby generating therapeutic strategies for Mets that will help prevent or improve cognitive impairment observed in AD patients (10). Although this relationship is supported by several evidence, the molecular mechanism of metabolic-cognitive syndrome is still being explored.

Currently, bioinformatics tools and software provides almost perfect and accurate comprehensive comprehensive analysis of cell metabolites (11). There is increasing evidence that the etiology of AD is closely related to the immune response (12). A network pharmacology analysis conducted by Liu (13) tentatively suggests that Yuanzhi powder may affect immune regulatory mechanisms AD treatment. A basic experiment elucidated that high sugar and high fat, as key factors of Mets, played a great role in the development of neuroinflammation and immunity (14). Currently, interdisciplinary research in neuroscience and immunology has linked nutritional excess with neuroinflammation (15). These findings highlight that immune mechanisms may play a key role in linking AD and MetS.

In recent years, integrated bioinformatics analysis has been used to identify new genes associated with various diseases, which may serve as biomarkers for diagnosis and prognosis. However, the common diagnosis and interlinked genes in MS and AD are unclear. Therefore, this study used bioinformatics methods to screen the biomarkers related to immune infiltration of the two, providing theoretical basis for diagnosis and treatment.

## Materials and methods

### Dataset acquisition, processing and differential expression analysis

The datasets we analyzed were obtained from the National Center for Biotechnology Information (NCBI) Gene Expression Omnibus (GEO) database (16), including the AD dataset GSE63060 (17), GSE63061 and one AD single-cell RNA-sequencing dataset GSE168522 (18); the same method was used to obtain the MetS dataset GSE (19). The software R (https://www.bioconductor.org/) was used for data analysis. Datasets were filtered, background corrected, log2 transformed and normalized. In addition, the datasets GSE63060 and GSE63061 were merged, and the merged data were batch corrected using the Combat method of the “sva” package. The |log2 Fold change (FC)| > 1 and p <0.05 were set as the criteria for identifying differentially expressed genes (DEGs) using the “limma” package in R.

### Single-cell sequencing quality control and dimensionality reduction

We downloaded the single-cell RNA sequencing dataset (GSE168522) from the GEO database. “Seurat” and “SingleR “ software packages were used to analyze the scRNA-seq dataset. The retained cells were those with ≤ 10% of mitochondrial genes and ≤ 3% of red blood cell genes. Simultaneously, we filtered out the cells with number of genes (nFeature RNA) ≤ 200 or ≥ 2000. Next, we performed dimensionality reduction and clustering and selected 3000 hypervariable genes. By combining with the elbow plot and selecting the inflection point and the PC with a smooth curve, we selected the first 10 dimensions for follow-up analysis and showed the dimension reduction effects of UMAP and tSNE. Then, we performed cell-related annotation through immune cell related maker (20). Finally, we visualized the expression of the hub gene in different immune cells using a violin diagram.

### Functional enrichment analysis

The Metascape database (https://metascape.org) was used for Gene Ontology (GO) (21)enrichment analysis and protein–protein interaction network construction (22). Kyoto Encyclopedia of Genes and Genomes (KEGG) enrichment analysis (23) was performed using the SangerBox database (http://sangerbox.com/Tool). In this study, we performed GO, KEGG and protein–protein interaction analyses on the up-regulated and down-regulated genes that were co-expressed by AD and MetS using the two databases. Simultaneously, we performed GO analysis on the key module genes obtained by weighted gene co-expression network analysis (WGCNA) analysis *via* the Metascape database.

### Weighted gene co-expression network analysis

To investigate the relationship between gene networks and diseases, we looked for the co-expressed gene modules (AD and MetS associated modules) with high biological significance using the algorithm of the WGCNA (24). The “WGCAN” package was used to construct the co-expression modules (25). We used genes with expression > 0 for further analysis to exclude outlier data. By setting the optimal soft threshold, we simultaneously identified the most relevant AD and MetS modules and the multi-co-expressed module genes for further analysis.

### Machine learning to screen candidate genes

Two machine learning algorithms, random forest (RF) (26) and least absolute shrinkage selection (LASSO) (27), were adopted to further filter candidate genes for AD and MetS diagnosis. RF is an ensemble prediction method that can handle a large number of input variables and evaluate the importance of variables. LASSO is a regression method that has shown superiority in evaluating high-dimensional data. We used the RF algorithm to initially screen diagnostic genes, with an importance score greater than 0. Among the obtained genes, the LASSO algorithm was utilized to further reduce the dimension to obtain the final diagnostic genes, as well as draw their respective ROC curves. We performed RF analysis and LASSO regression with the R packages “random forest” and “glmnet” (28).

### Evaluation of candidate gene diagnostic value

The eXtreme Gradient Boosting (XGBoost) (29), a commonly used supervised integrated learning algorithm, has strong scalability and convenient features to facilitate model visualization and optimization. We used the XGBoost to build candidate gene models with the training set (GSE63060) and evaluated them on the validation set (GSE63061). Subsequently, the receiver operating characteristic (ROC), precision-recall (PR) curves, and area under the curve (AUC) were drawn to evaluate the diagnostic efficacy of the model. It was verified in MetS.

### Immune infiltration analysis

The CIBERSORT deconvolution algorithm (26) is a machine learning method based on linear support vector regression, which is a calculation method used to evaluate the percentage of 22 immune cells in tissues or cells. The experiment was based on R and linked to CIBERSORT deconvolution method to simulate the transcription characteristic matrix of 22 kinds of immune cells, such as B cells, plasma cells, T cells, natural killer cells, monocytes, macrophages, dendritic cells, mast cells, eosinophils, and neutrophils. We compared the immune cell infiltration of peripheral blood mononuclear cells (PBMC) samples from the disease group with normal samples. Meanwhile, the relationship between the hub gene and immune cells in AD and MetS was explored.

The hallmark gene set was downloaded from Molecular Signature Database (MSigDB) (27), and the metabolic related pathways were analyzed by single sample gene set enrichment analysis (ssGSEA) of the hub genes. The ssGSEA was an extension algorithm of the GSEA approach. Finally, we performed a correlation analysis of immune infiltration and metabolism in AD and MetS.

### Statistical analysis

All statistical tests were implemented using the R software version 4.1.2. The Wilcoxon or Student’s t-test was utilized to analyze the difference between the two groups. The correlation between the variables was determined using the Pearson’s or Spearman’s correlation test. Statistical significance was set at a two-tailed p < 0.05.

## Results

### Identification of DEGs in AD and MetS

**Figure 1:**
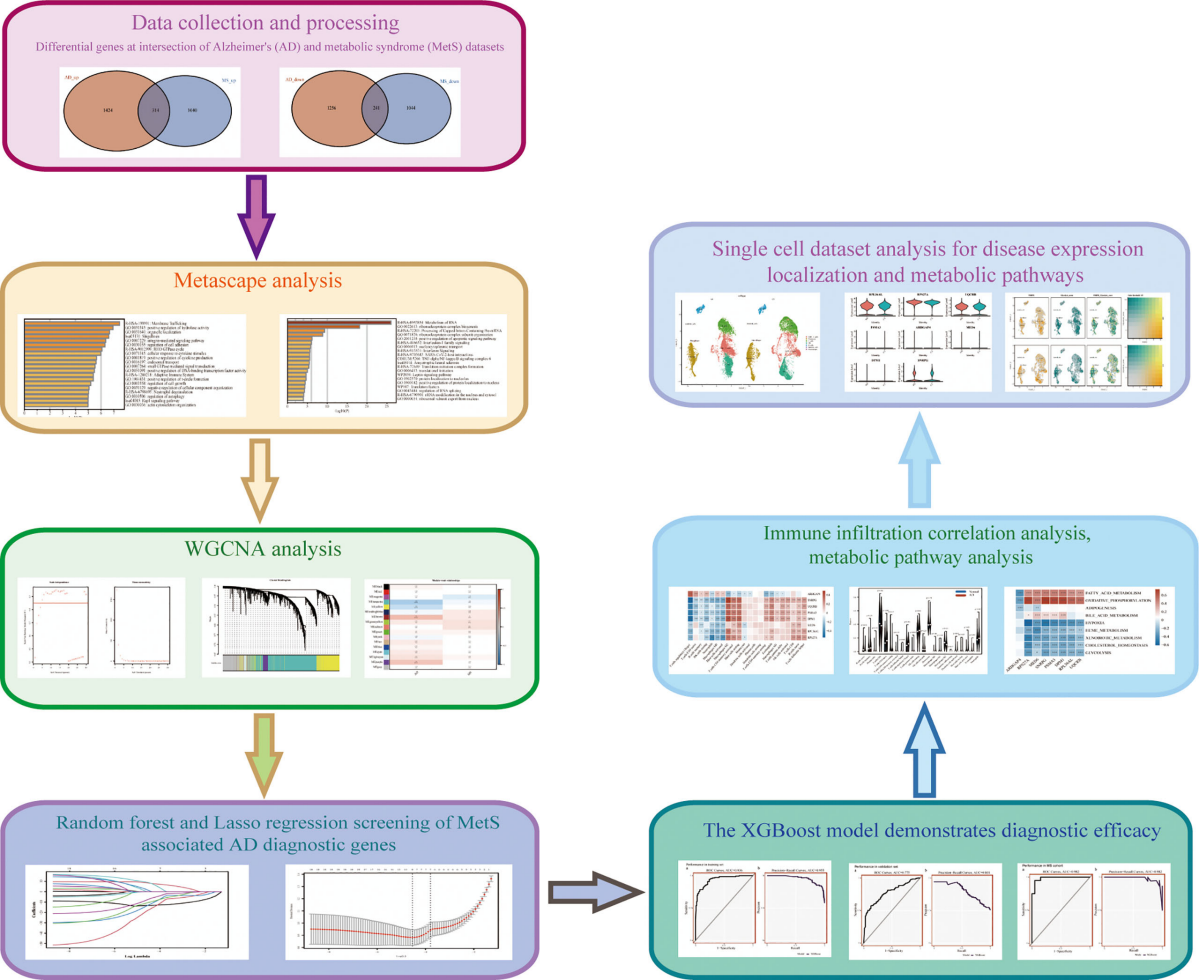
Research technology flow chart.

**Figure 2:**
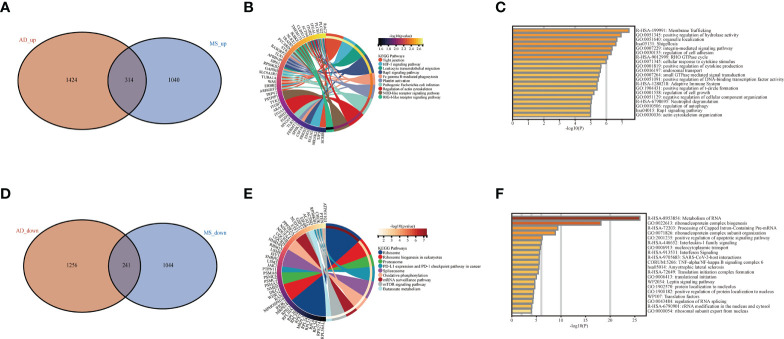
Difference and enrichment analysis of AD and MS patients. **(A)** The intersection of AD up-regulated DEGs and MetS up-regulated DEGs. **(B)** KEGG analysis of up-regulated intersection genes. **(C)** GO analysis of up-regulated intersection genes. **(D)** The intersection of AD down-regulated DEGs and MetS down-regulated DEGs. **(E)** KEGG analysis of down-regulated intersection genes. **(F)** GO analysis of down-regulated intersection genes.

The research flowchart of is shown in **Figure 1**. The principal component analysis (PCA) was used to visualize the distribution of these samples prior to and after correcting batch effect. In addition, before data correction and normalization, we performed PCA on the three datasets (GSE63060, GSE63061 and GSE8895) (**Supplementary Figures S1E, F**). After standardizing the dataset results, 3235 DEGs (1738 upregulated and 1479 downregulated) were identified in AD, while 2639 DEGs (1354 upregulated and 1285 downregulated) were identified in MetS. In addition, a Venn diagram analysis was performed to evaluate the common DEGs between AD and MetS, showing that 314 and 241 overlapping DEGs were identified in the up-regulated and down-regulated DEGs, respectively (**Figures 2A, D**).

### Enrichment analysis of AD and MetS co-upregulated- and co-down-regulated genes

In order to explore the biological functions and pathways of the identified overlapping DEGs, we performed GO and KEGG analyses, which were plotted through bar diagram and cnetplots. KEGG analyses results showed that the up-regulated DEGs were mainly enriched in tight junction, HIF-1 signaling pathway and leukocyte transendothelial migration, the down-regulated DEGs were enriched in the ribosome, spliceosome and mTOR signaling pathway (**Figures 2B, E** **)**. Based on the GO analysis, we could see the up-regulated DEGs were involved in membrane trafficking and positive regulation of hydrolase activity, whereas the down-regulated DEGs were involved in metabolism of RNA ribonucleoprotein complex biogenesis and positive regulation of apoptotic signaling pathway (**Figures 2C, F**).

### Co-expressed modular genes and enrichment in AD and MetS

**Figure 3:**
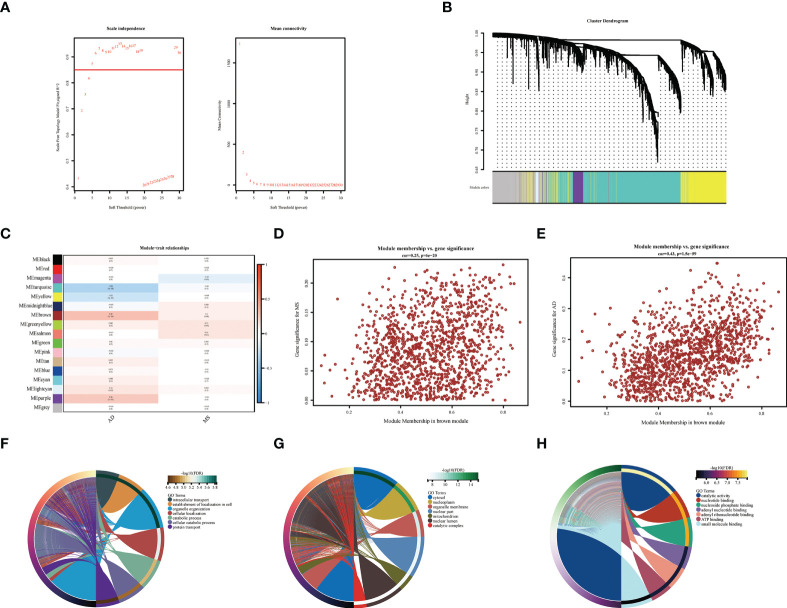
WGCNA co-expression and enrichment analysis in AD and MS patients. **(A)** Analysis of network topology for various soft-thresholding powers. **(B)** The cluster dendrogram of co-expression genes in AD and MetS. **(C)** Module–trait relationships in AD and MetS. Each cell contains the corresponding correlation and p-value. **(D)** The correlation between genes and AD in the brown module. **(E)** The correlation between genes and MetS in the brown module. **(F)** The BP in GO analysis of co-expression genes in AD and MetS. **(G)** The CC in GO analysis of co-expression genes in AD and MetS. **(H)** The MF in GO analysis of co-expression genes in AD and MetS.

Co-expression analysis was employed to construct the co-expression network. In our study, cluster analysis was performed with the “flash clust” function. With a threshold set to 20, 13 outlier samples were detected and removed, and 51 samples were retained (**Supplementary Figures S1G, H**). The “pick Soft Threshold” function in the “WGCNA” package to filter out the power parameters from 1 to 30. A power of β = 5 was chosen as the most appropriate soft threshold to guarantee the scale-free network (**Figure 3A**). The “cutree” dynamic and module eigengenes functions to construct cluster diagram (**Figure 3B**), A total of 17 modules consisting of genes with similar co-expression traits were obtained. Then, a heat map about module–trait relationships was mapped according to the Spearman correlation coefficient to evaluate the association between each module and the disease (**Figure 3C**). The modules “brown” indicates a high connection between AD and MetS (AD: r = 0.29, p = 1e−06; MetS: r = 0.1, p =0.08). The brown module contains the positively correlated genes of AD and the negatively correlated genes of MetS (**Figures 3D, E**). GO analysis of this brown module gene for AD and MetS was performed through the Metascape website. The results showed that it was mainly enriched in intracellular transport, establishment of localization in cell and organelle organization in BP, cytosol, nucleoplasm, organelle membrane in CC and catalytic activity, nucleotide binding and nucleotide phosphate binding in MF (**Figures 3F–H** **)**.

### Identification of candidate central genes in AD and MetS using machine learning

**Figure 4:**
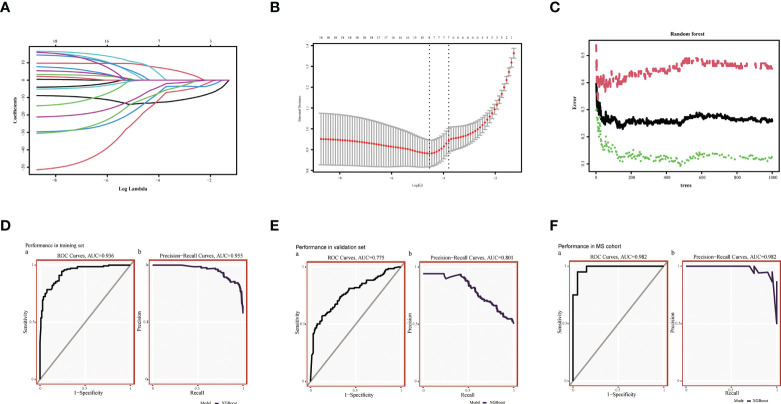
Machine Learning Screening Genes and Modeling. **(A)** LASSO coefficient profiles of candidate genes. **(B)** Cross-validation to select the optimal tuning parameter log (Lambda) in LASSO regression analysis. **(C)** RF coefficient profiles of candidate genes. **(D)** XGBost modeling in AD training set. **(E)** Validate through the AD validation set. **(F)** Validate with MetS dataset.

Using the RF method, we initially screened out 30 diagnostic genes (**Figure 4C**), and then used LASSO to further reduce the dimensionality to obtain the final eight diagnostic genes (**Figures 4A–C** **)**, namely *ARHGAP4*, *SNRPG*, *UQCRB*, *PSMA3*, *DPM1*, *MED6*, *RPL36AL*, *RPS27A*. We further evaluated the diagnostic values of these genes. The AUC values of ROC curves were 0.716 of *ARHGAP4* (**Supplementary Figure S2A**), 0.725 of *DPM1* (**Supplementary Figure S2B**), 0.774 of *MED6* (**Supplementary Figure S2C**), 0.774 of PSM3 (**Supplementary Figure S2D**), 0.841 of *RPL36AL* (**Supplementary Figure S2E**), 0.809 of *RPS27A* (**Supplementary Figure S2F**), 0.720 of *SNRPG* (**Supplementary Figure S2G**) and 0.775 of *UQCRB* (**Supplementary Figure S2H**). We found that they all had high accuracy with AUC >0.7, revealing the predictive efficacy of all eight gene signatures.

We used the XGBoost to build candidate gene models with the training set **(**GSE63060**)**, evaluated them on the validation set **(**GSE63061**).** In GSE63060, the VUC of ROC was 0.936 and PR was 0.955 **(** **Figure 4D** **)**. For GSE63061, ROC was 0.775 and PR was 0.801 (**Figure 4E**), which illustrated the diagnostic efficacy of the model. Similarly, it was verified in MetS, indicating that the model is also applicable and effective in MetS, with a ROC of 0.982 and PR of 0.982 (**Figure 4F**).

### Immune infiltration analysis in AD and MetS patients

**Figure 5:**
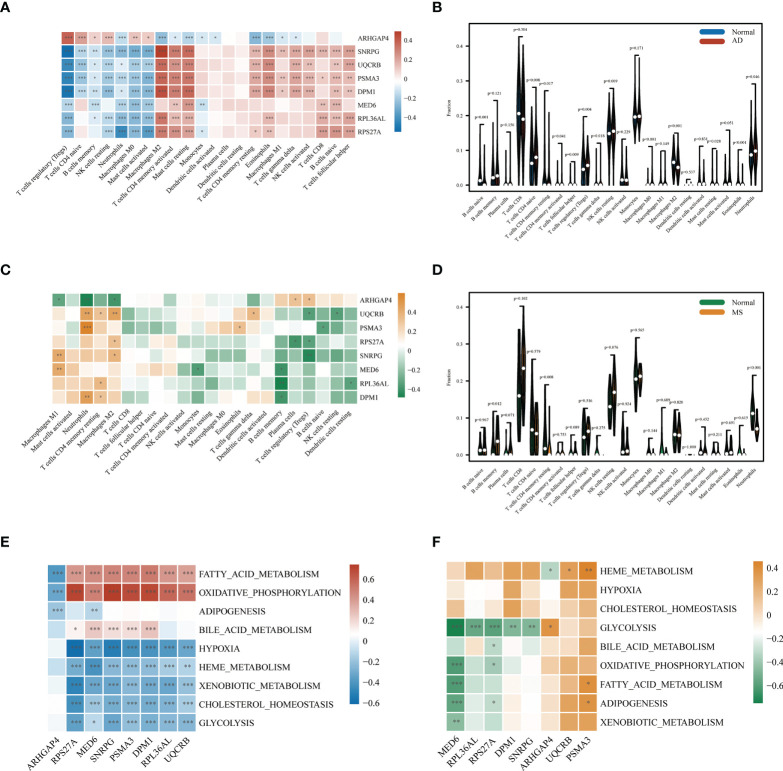
Correlation of AD and MS patients with immune cells and metabolic signaling pathways. **(A)** Immune infiltration analysis of eight candidate genes in AD. **(B)** Comparison of immune cell infiltration between AD and CT samples. **(C)** Immune infiltration analysis of eight candidate genes in MetS. **(D)** Comparison of immune cell infiltration between MetS and CT samples. **(E)** Metabolic pathway analysis of eight candidate genes in AD. **(F)** Metabolic pathway analysis of eight candidate genes in MetS. *P < 0.05, **P < 0.01, ***P <0.001.

We performed immune infiltration analysis on these eight key genes, and the analysis results revealed that seven genes (*SNRPG*, *UQCRB*, *PSMA3*, *DPM1*, *MED6*, *RPL36AL*, *RPS27A*) had significantly lower levels of activated regulatory T cells (Tregs), CD4 naïve cells, memory B cells, resting NK cells, neutrophils, macrophages M0, and mast cells than *ARHGAP4* in AD patients (p<0.01). In contrast, the levels of macrophages M2, T cells, mast cells resting, dendritic cells activated, CD4 memory resting T cells, eosinophils, macrophages M1, gamma delta T cells, CD8 NK cells, naïve B cells and follicular helper T cells were significantly higher than *ARHGAP4* in AD patients **(** **Figure 5A** **)**. However, seven genes (*SNRPG*, *UQCRB*, *PSMA3*, *DPM1*, *MED6*, *RPL36AL*, *RPS27A*) had a higher levels of macrophages M1, neutrophils, CD4 memory resting T cells and macrophages M2, as well as lower level of memory B cells, regulatory T cells (Tregs) and resting dendritic cells than *ARHGAP4* (p<0.01) **(** **Figure 5C** **)**. The correlation between the immune cell contents patients and control in AD or MetS was also calculated. The patients with AD showed a higher proportion of macrophages M0, and a lower proportion of B cells naïve, macrophages M2 and eosinophils **(** **Figure 5B** **).** Patients in MetS showed a lower proportion of eosinophils than control **(** **Figure 5D** **)**. According to ssGSEA metabolic analysis, the metabolic pathways of hub genes in AD dataset were mainly clustered in glycolysis, hypoxia and oxidative phosphorylation adiposis **(** **Figure 5E** **),** and the metabolic pathways of Hub gene in MetS were mainly clustered in glycolysis and heme metabolism **(** **Figure 5F** **)**.

### Single-cell sequencing analysis in AD and normal patients

**Figure 6:**
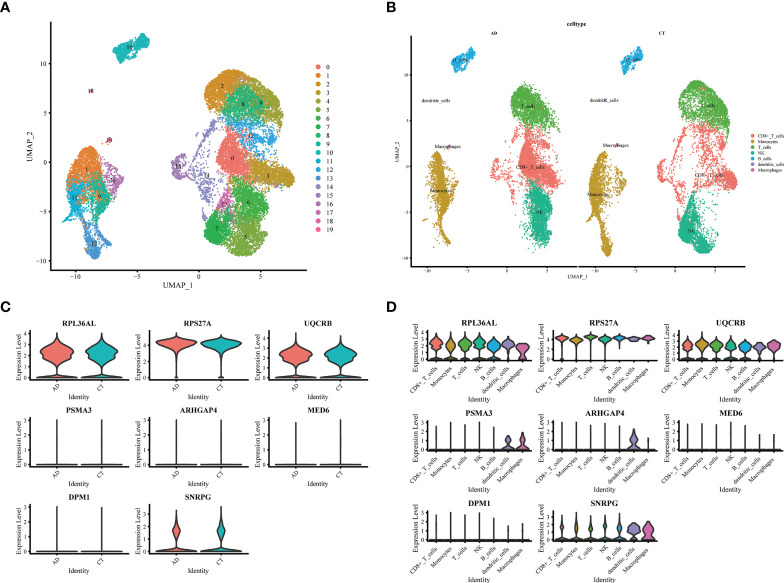
Expression of 8 model genes in immune subsets of AD and normal patients. **(A)** UMAP display of single cell grouping in patients with AD. **(B)** AD and normal patients are divided into 7 immune cell subsets. **(C)** Violin pictures show the expression of model genes in normal and AD patients. **(D)** The violin picture shows the expression of model genes in immune cells.

**Figure 7:**
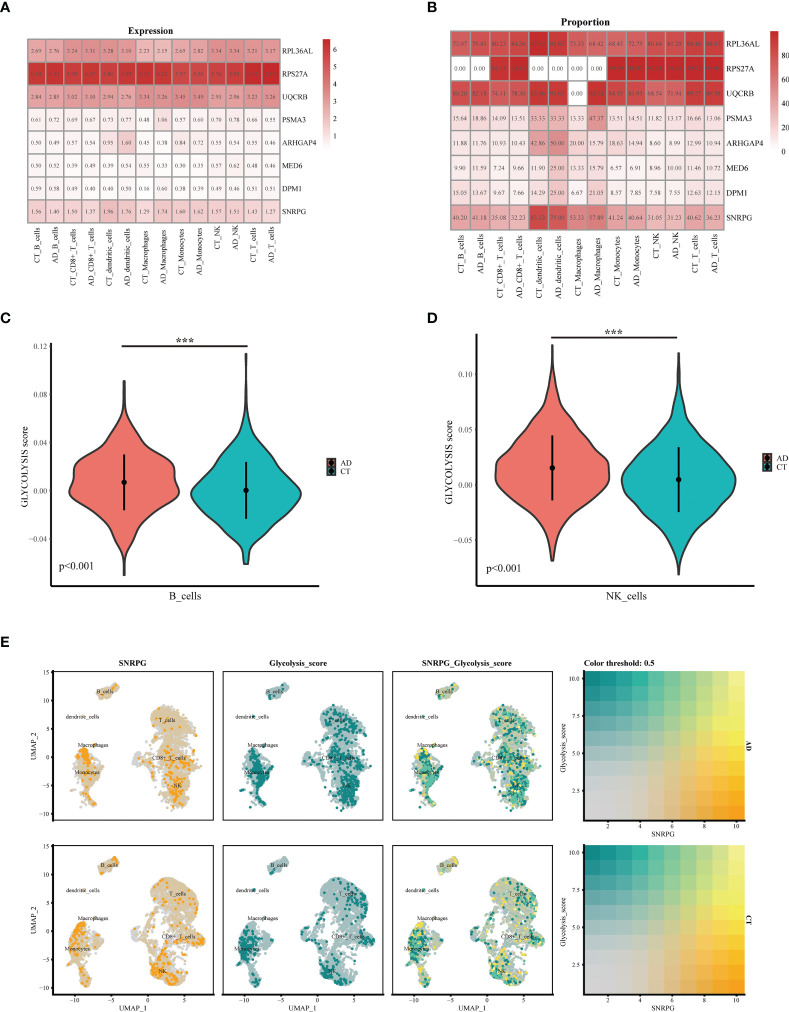
Expression and co-localization of key genes in immune cells of AD patients. **(A, B)** Expression of different genes in immune cells of AD and normal patients. **(C)** The violin shows the difference in the fraction of glucose metabolism in B cells between normal patients and AD patients. **(D)** The violin shows the difference in the fraction of glucose metabolism in NK cells between normal patients and AD patients. **(E)** Colocalization of glucose metabolism and SNRPG in AD patients and normal patients, respectively. ***p<0.001.

We downloaded the single-cell RNA sequencing dataset (GSE168522) from the GEO database and selected a healthy and AD patient in the dataset as Seurat object for analysis. First, we conducted data quality control. We retained cells with less than 10% of mitochondrial genes and less than 3% of red blood cells. Cells with a gene number (nFeature RNA) greater than 2000 or less than 200 were filtered out (**Supplementary Figures S3A, B** **)**. We identified 3000 hypervariable genes and marked the 10 most important genes. All hypervariable genes were highlighted in red as shown in **Supplementary Figure S3C**. T-SNE algorithm was used to cluster all cells. The cells could be divided into 19 categories (**Supplementary Figure S3D**). Uniform Manifold Approximation and Projection (UMAP) was used for non-linear dimension reduction. The “FindCluster” function was used to cluster cells, obtaining 19 clusters (**Figure 6A**). The result revealed increased percentage of monocytes clusters, B cells, T cells, CD8+_Tcells and NK in the AD group (**Figure 6B**). The expression of cell type marker genes is shown in the dot plot (**Supplementary Figure S3E**). Next, we performed cell related annotation through immune cell between AD and CT groups, which showed that *RPL36AL*, *RPS27A*, *UQCRB* and SNRPC were highly expressed in the two groups, while the remaining four genes were less expressed (**Figure 6C**). Finally, we visualized the expression of eight hub genes in different immune cells by violin diagram. *RPL36AL*, *RPS27A*, *UQCRB* and SNRPC were annotated in all seven cell groups, *PSMA3* was by dendritic cells and macrophages, *ARHGAP4* was only by dendritic cells, *MED6* and *DPM1* were hardly annotated. Then we performed cell ratio and expression analysis, *RPL36AL*, *RPS27A*, *UQCRB* and SNRPC were highly expressed in the seven cells types of all samples (**Figure 7A**), *RPL36AL*, *UQCRB* and SNRPC had a high expression rate, *RPS27A* had a high expression rate in CD8_T_cells, NK cells and T cells in two samples (**Figure 7B**). The results were basically consistent with the **Figure 6D** we analyzed before. Through ssGSEA metabolic pathway analysis, we found that the glucose metabolism scores of B cells and NK cells in normal and AD were different (**Figure 7C, D** **)**. In the two cell populations, the glucose metabolism fraction in AD was lower than that in the normal group, and the analysis showed that *SNRPG* participated in this important pathway (**Figure 7E**).

## Discussion

Candidate therapies that effectively target the core pathology of AD have achieved disappointing results in clinical trials, and this failure may be attributed to late intervention in the course of the disease (28). There is a growing consensus that therapeutic intervention must be started at the early stage of the disease (preclinical or prodromal symptoms) to make meaningful disease changes in AD (1). Moreover, studies have shown that multiple factors drive the cell phase of AD. For example, early impaired brain metabolism seems to play an important role in cognitive decline. Specifically, defects in glucose metabolism in the frontal and temporal parietal lobes may contribute to disease progression (30). Insulin resistance (IR) was the main feature of MetS. Because it could increase the accumulation of and NP, it was thought to play an important role in the metabolism of tau protein, which affects the development of AD. In addition, IR was an important link between MetS and MCI (2, 31). Therefore, we used machine learning method to identify the key genes of AD in PBMC for the identification of early patients.

Although considerable literature has examined the link between AD and MetS, few studies have explored the common diagnostic effector genes of the two diseases, as well as the correlation between these genes and immune cells. To explore the common diagnostic effector genes of the two diseases, we used WGCNA to obtain the co-expression module (brown module), and the biological functions of this module genes were clustered in cell localization, transport and catabolism. Furthermore, intracellular transport played an irreplaceable role in the molecular mechanism of AD. For instance, intracellular transport changed in the expression of app and tau through intracellular transport, leading to cognitive decline and neurodegeneration in AD (32). High levels of circulating lipid and glucose imbalance can lead to high levels of oxidative metabolism, leading to neuronal damage (33). These studies were consistent with our findings. Moreover, our study explored genes in which AD and MetS have a common diagnostic effect.

We obtained eight co-effect genes, namely *ARHGAP4*, *SNRPG*, *UQCRB*, *PSMA3*, *DPM1*, *MED6*, *RPL36AL* and *RPS27A* through machine learning methods. ROC analysis showed that they had good prediction effect. It could be seen from the immune infiltration analysis that the four genes (*RPL36AL*, *RPS27A*, *UQCRB* and SNRPC) were highly expressed in different immune cell subpopulations. Ribosomal Protein L36a Like (*RPL36AL*) is a Protein Coding gene that mainly exists at the E-site in human ribosomes and can be crosslinked *in situ* with the CCA end of p-tRNA (34, 35). Previous studies have shown that *RPL36AL* acts as an immune related gene in AD, but its mechanism has not been elucidated (35, 36). In the immune infiltration analysis, *RPL36AL* was mainly on B cells. Single-cell RNA sequencing reveals significant decrease in B cells detected in blood of AD patients, and similar studies have previously yielded similar results (18). Ribosomal protein S27A (*RPS27A*),as a ribosomal protein, was mainly involved in the functional role of ribosomal biogenesis and post-translational modification (37). It can perform both synthetic ribosomal and *in vitro* ribosomal functions (38, 39), and overexpression in multiple malignancies, such as leukemia (40). *RPS27A* might act as a controller of microglia activation in triggering neurodegenerative diseases (41). Small Nuclear Ribonucleoprotein Polypeptide C (SNRPC), was a protein coding gene (42). Relevant have shown that SNRPC was the main causative gene of MCI and AD (43). MetS was a risk factor for AD (44). One study found that tau phosphorylation may accelerate psychosis in AD (45). These results suggested that the Hub gene we are looking for may be involved in the occurrence and development of AD and MetS diseases. There are very few studies on the above three genes in AD and MetS, so our analysis can only be used as a preliminary reference, so follow-up experiments are needed to further confirm.

The brain was the most abundant organ of human energy metabolism. Although the adult brain accounted for 2% of the total body weight, it used 25% of the whole body’s glucose during rest and wakefulness (46), which makes it vulnerable to impaired energy metabolism. Accumulated evidence indicated that AD was an age-related metabolic neurodegenerative disease (47). One of the pathophysiological features of AD was impaired cerebral glucose metabolism, which occurred long before cognitive impairment and pathological changed, and this prodromal period could last up to 10 years (48–50). Research confirmed diabetes can increase the risk of AD, and even the use of hypoglycemic drugs cannot reduce the risk (51), which can be increased two to threefold by previous study, which is not related to the risk of vascular dementia (52–55). Recombinant interferon γ the metabolic enhancement of treatment reversed the glycolytic metabolism and inflammatory functional defects of microglia, thereby alleviating the AD pathology of 5xfad mice (56). Through metabolic correlation analysis, we proved the correlation of hub gene in glucose metabolism related pathways of AD and MetS. Next, we will continue to further explore the mechanism of glucose metabolism for AD in animal models.

The XGBoost machine learning model further improves the diagnostic value of eight genes in AD and MetS, which is helpful for the early diagnosis of patients through PBMC. However, this study still has some limitations. First, the available clinical information in public databases is limited, which may lead to biased results. Second, further *in vitro* experiments are needed to better understand the common mechanism of MetS and AD regulation. Finally, more data sets or clinical prospective studies are needed to validate the identified diagnostic genes. To alidate the experimental findings, it is necessary to collect more clinical samples for clinical validation, as well as to perform modeling of rat models and knock out some key genes.

## Conclusion

Our study provides key co-diagnostic effector genes for AD and MetS patients, while revealing that disease co-involvement genes are associated with diverse immune cells. Glucose metabolism-related pathways may be the common mechanism of AD and MetS, and glucose metabolism may act on AD patients through NK cells and B cells. Meanwhile, we found that the gene *SNRPG* may act as a key gene related to glucose metabolism in AD patients.

## Data availability statement

The original contributions presented in the study are included in the article/**Supplementary Material**. Further inquiries can be directed to the corresponding author.

## Ethics statement

Ethical review and approval was not required for the study on human participants in accordance with the local legislation and institutional requirements. Written informed consent for participation was not required for this study in accordance with the national legislation and the institutional requirements.

## Author contributions

JL, YZ and TL participated in the manuscript of the article and the conception of the article, ZW, TL and RL edited the picture and data analysis, ML, XY and JZ participated in the review of the article. SD embellished the article and revised the grammar of the article. QL modified the article.

## Funding

This study was supported by Liuzhou City’s Top Ten Hundred Talents Project, Liuzhou Science and Technology Project (Grant No. 2021CBC0126 and 2021CBC0123), Guangxi Zhuang Autonomous Region Health and Family Planning Commission Projects (Z20210561, Z20210903), and Liuzhou Science and Technology Plan Projects (2021CBC0121, 2021CBC0128).

## Acknowledgments

We thank Dr.Jianming Zeng(University of Macau), and all the members of his bioinformatics team, biotrainee, for generously sharing their experience and codes. We thank composer Samshin for some guidance. We would like to thank Editage (www.editage.cn) for English language editing.

## Conflict of interest

The authors declare that the research was conducted in the absence of any commercial or financial relationships that could be construed as a potential conflict of interest.

## Publisher’s note

All claims expressed in this article are solely those of the authors and do not necessarily represent those of their affiliated organizations, or those of the publisher, the editors and the reviewers. Any product that may be evaluated in this article, or claim that may be made by its manufacturer, is not guaranteed or endorsed by the publisher.

## Supplementary material

Supplementary Figure 1 Enrichment analysis and dataset quality control. **(A–D)** Enrichment and protein interaction analysis of co-up- and down-regulated genes in AD and MetS patients. **(E, F)** PCA between integrated datasets after de-batching. **(G)** Before clustering of WGCNA analysis **(H)** After clustering of WGCNA analysis.

Supplementary Figure 2 Receiver operating characteristic (ROC) curves of 8 candidate genes. **(A–H)** ROC analysis of eight candidate genes

Supplementary Figure 3 Single-cell quality control and annotation. **(A)** The genes (features), counts, and mitochondrial gene percentage before quality control. **(B)** The genes (features), counts, and mitochondrial gene percentage after quality control. **(C)** Correlation between genes and counts on the left side. Highly variable genes (HVGs) were colored in red, and the top 10 HVGs were labeled on the right side. **(D)** All cells were clustered using t-SNE algorithm. This experiment could be divided into 19 clusters. **(E)** Dot plot of cell type marker genes of top differentially expressed genes for each T and NK cluster.

## Abbreviations

AD, Alzheimer’s disease; MetS, Metabolic syndrome; XGBoost, eXtreme Gradient Boosting; PBMCs, peripheral blood mononuclear cells; scRNA, single-cell RNA; GEO, Gene Expression Omnibus; WGCNA, weighted gene co-expression network analysis; RF; Random Forest.
