# Supplementary figures and images for: Identification of diagnostic genes for both Alzheimer’s disease and Metabolic syndrome by the machine learning algorithm
Source: Front Immunol. 2022 Nov 2;13:1037318. doi: 10.3389/fimmu.2022.1037318 (PMC9667080; doi:10.3389/fimmu.2022.1037318)

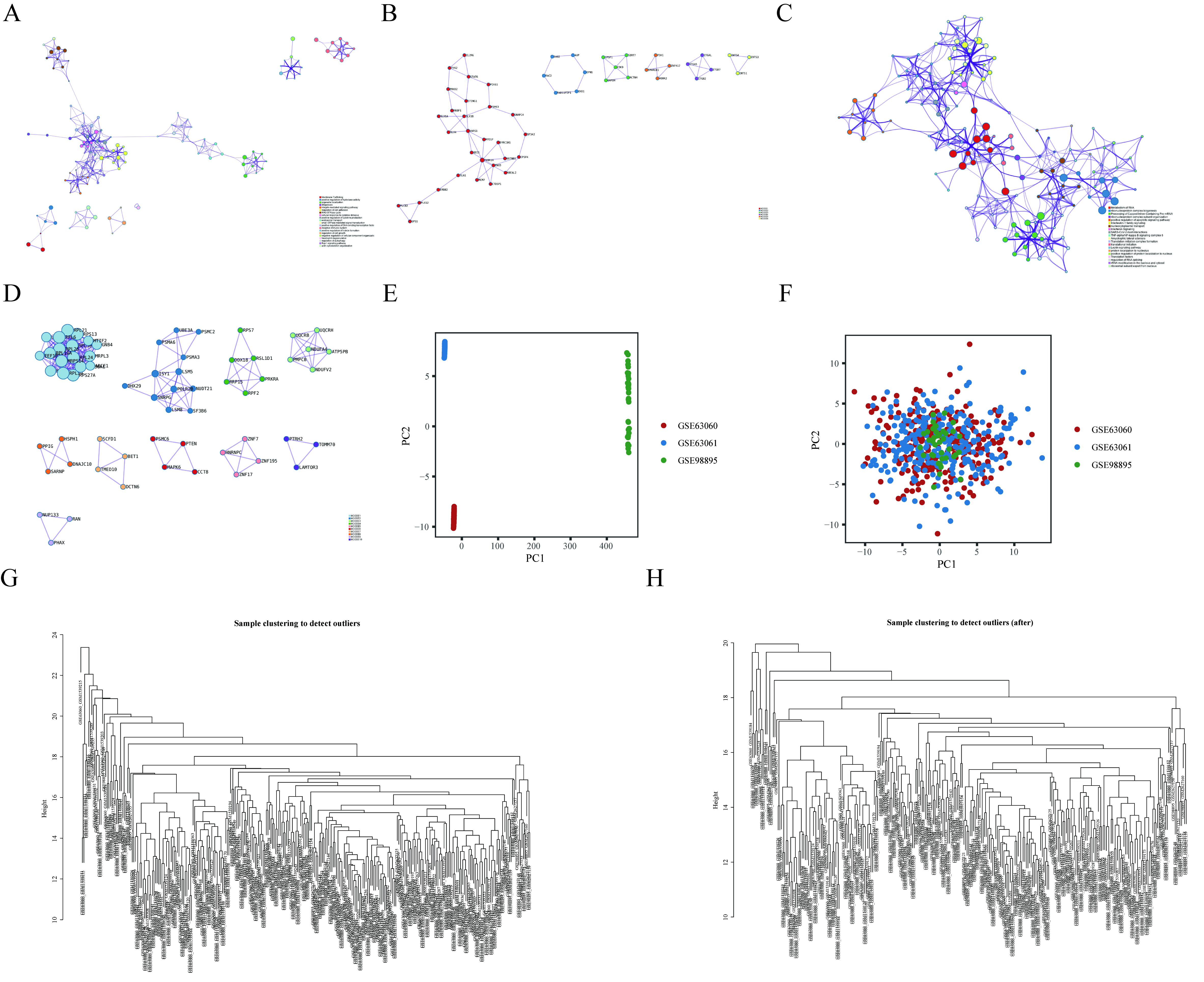

Supplement: Supplementary Figure 1 — Enrichment analysis and dataset quality control. (A–D) Enrichment and protein interaction analysis of co-up- and down-regulated genes in AD and MetS patients. (E, F) PCA between integrated datasets after de-batching. (G) Before clustering of WGCNA analysis (H) After clustering of WGCNA analysis. [file Image_1.tif]

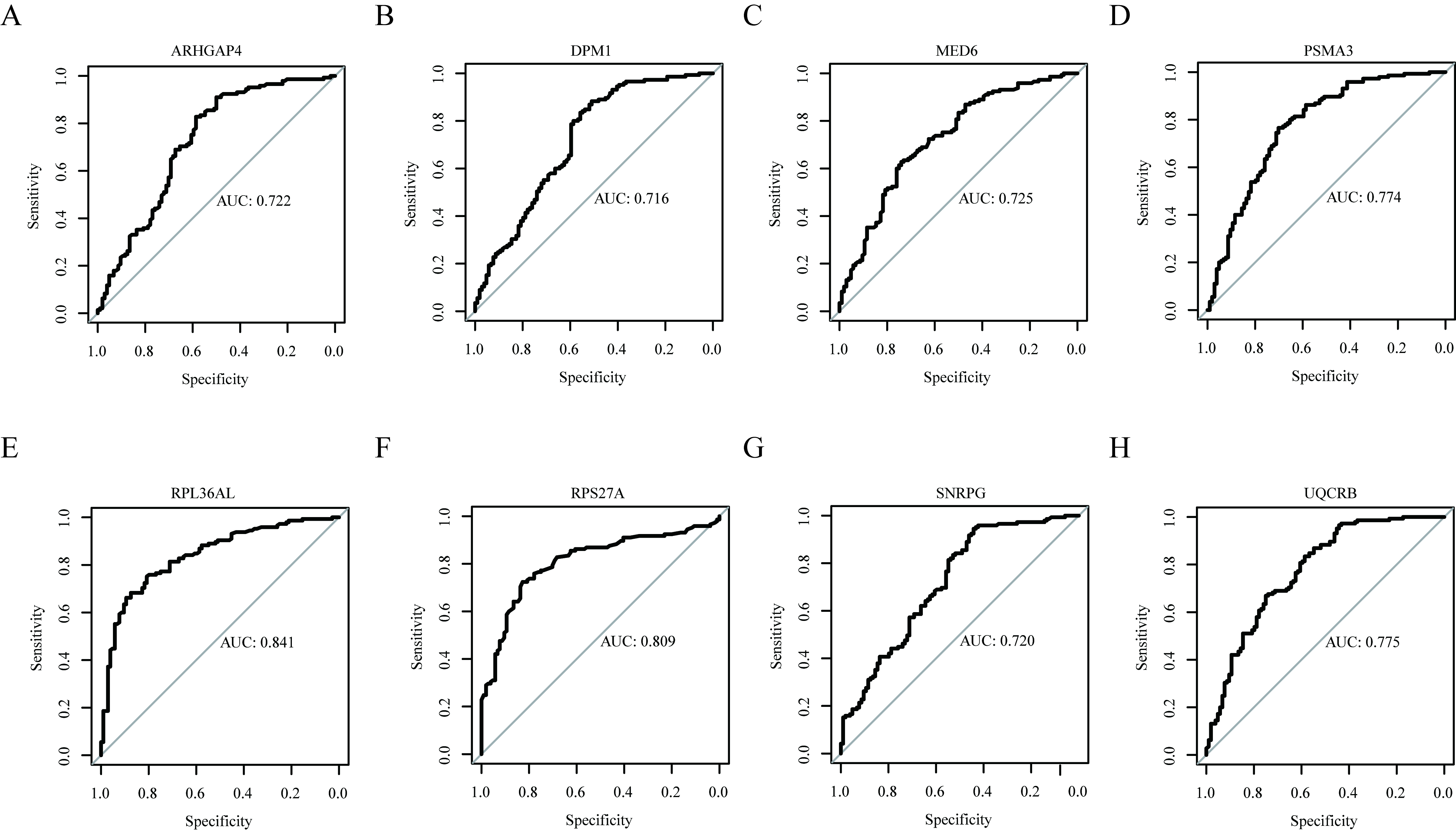

Supplement: Supplementary Figure 2 — Receiver operating characteristic (ROC) curves of 8 candidate genes. (A–H) ROC analysis of eight candidate genes [file Image_2.tif]

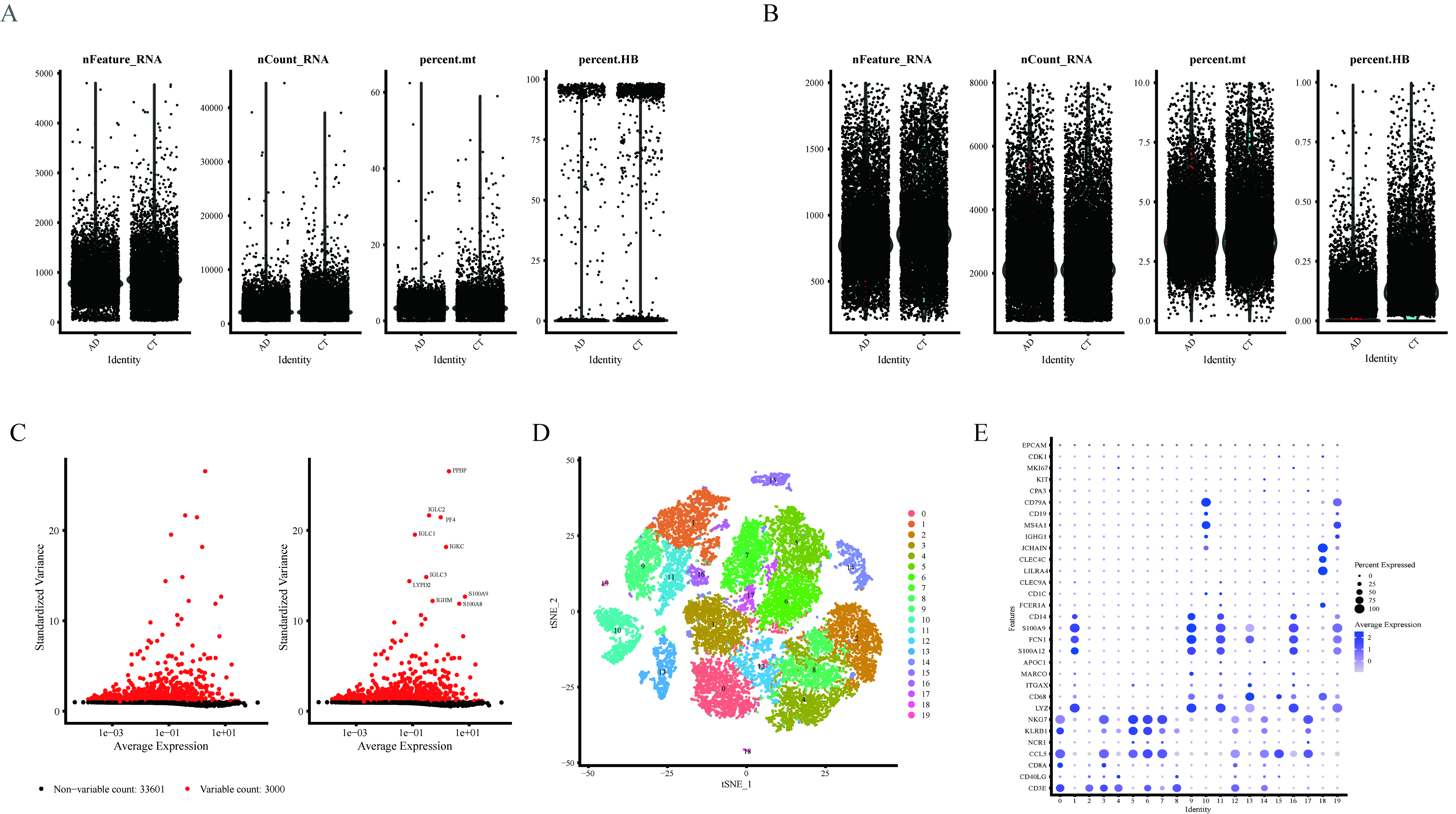

Supplement: Supplementary Figure 3 — Single-cell quality control and annotation. (A) The genes (features), counts, and mitochondrial gene percentage before quality control. (B) The genes (features), counts, and mitochondrial gene percentage after quality control. (C) Correlation between genes and counts on the left side. Highly variable genes (HVGs) were colored in red, and the top 10 HVGs were labeled on the right side. (D) All cells were clustered using t-SNE algorithm. This experiment could be divided into 19 clusters. (E) Dot plot of cell type marker genes of top differentially expressed genes for each T and NK cluster. [file Image_3.tif]
